# Insecticide resistance in malaria-transmitting mosquitoes in Zimbabwe: a review

**DOI:** 10.1186/s40249-015-0076-7

**Published:** 2015-10-26

**Authors:** White Soko, Moses J. Chimbari, Samson Mukaratirwa

**Affiliations:** School of Nursing and Public Health, University of KwaZulu-Natal, Howard Campus, Durban, 4001 South Africa; College of Health Sciences, University of KwaZulu-Natal, Durban, South Africa; School of Life Sciences, University of KwaZulu-Natal, KwaZulu-Natal, South Africa; Ministry of Health and Child Care, National Institute of Health Research, P.O. Box CY 573, Causeway, Zimbabwe

**Keywords:** Malaria, Mosquitoes, Vector control, Insecticide resistance, Insecticide sensitivity, Zimbabwe

## Abstract

Malaria is a global public health problem, with about 3.2 billion people at risk of infection. The populations at risk mainly reside in Africa, Asia and America, with African populations accounting for the largest burden of the disease. In 2013, close to 198 million malaria cases were reported, leading to 584,000 deaths. Much (90 %) of the mortality rates were recorded from the World Health Organization (WHO) database in the African region and 78 % of these occurred in children under the age of five. In Zimbabwe, approximately half of the population is at risk of infection with malaria.

Insecticide residual spraying (IRS) has been documented as an effective way to control malaria and has been adopted globally by the WHO and national governments. However, both insecticide resistance and climate change threaten to reverse the progress made by IRS in malaria control. Resistance has been reported in all four classes of insecticides approved by the WHO for vector control intervention. Variability of environmental temperature is suspected to complicate the situation through alteration in the genetic structure, and enzyme and protein profiles of mosquitoes. In Zimbabwe, little research has been done on the interaction between climate change, temperature variability and insecticide resistance in malarial mosquitoes over time. Such information is important for informing policies on insecticide selection for IRS.

We reviewed literature on insecticide sensitivity among malarial mosquitoes in Zimbabwe from 1972 to 2014. International peer-reviewed articles on insecticide sensitivity in Zimbabwe, published in English in this time period, were searched using MEDLINE® (PubMed), Google Scholar, Google and grey literature. Eight publications were eligible for the present study, with one of the articles being a review paper. Six articles covered insecticide resistance, while the other two articles, published in 2000, were about the absence of resistance. Contradicting resistance results were reported in 2014.

The insecticide sensitivity status and distribution of insecticide resistance in mosquitoes are still under debate in Zimbabwe, as studies report differing results. The resistance trend in Zimbabwe is characterised by fluctuations in the status of the sensitivity of existing insecticides. Inconsistencies in data collection methods may be responsible for the inconsistencies in the results. None of the studies have determined a link between climate/temperature variability and insecticide resistance as yet. The current insecticide sensitivity status of mosquitoes still needs to be verified.

## Introduction

Malaria is a vector-borne disease endemic in tropical and subtropical areas [1]. Close to 3.2 billion people are at risk of infection [2]. In 2013, an estimated 584,000 deaths from malaria were reported worldwide, with most (90 %) of the deaths occurring in the African region (as recorded in the World Health Organization [WHO] database). Seventy-eight per cent (78 %) of these deaths occurred in children under five years of age [2].

Zimbabwe has a population of about 13 million [3], with half of that population living in malaria-endemic areas [4, 5]. *Anopheles gambiae* complex, *Anopheles arabiensis* Patton and *Anopheles gambiae sensu stricto* Giles mosquitoes are responsible for the transmission of malaria in the country [6, 7], with *An. arabiensis* mosquitoes responsible for the majority of the transmission [7, 8]. The advent of climate change, especially increases in temperature, threatens to complicate the situation by extending the geographical distribution of malaria globally [9], in parts of Europe [10], Asia [11] and Africa [12–14].

Insecticide residual spraying (IRS) and long-lasting insecticide treated nets (LLINs) are the major intervention strategies aimed at interrupting malaria transmission [15]. In Zimbabwe, malaria case management, vector control using IRS and LLINs, and health education form the vanguard of the malaria control programme [4]. However, the WHO [16] has noted that insecticide resistance could derail disease control, with Krostad [17] expressing the same sentiment, saying that insecticide resistance was threatening to reverse the progress made by IRS in malaria control thus far.

Insecticide resistance involves changes in one or more genes, leading to the reduction in insecticide sensitivity of an insect population. This is manifested in an insecticide’s repeated failure to achieve the projected level of control when used following the recommendations for that species [18]. The changes leading to resistance may not only be genetic, but also enzymatic; at times genetically related and at times not [19–21]. Wood et al. [22] indicated that insecticide resistance could happen due to selection pressure and increasing mutation rates.

Insect resistance to dichlorodiphenyltrichloroethane (DDT) emerged in the 1940s, with the first conclusive study being conducted on the *Culex molestus* mosquitoes in 1947 in Italy. Insecticide resistance was also reported among *Anopheles sacharovi* mosquitoes in Greece in 1951 [23]. In 1955, it was reported in the *An. gambiae* species in Nigeria [24]. Thereafter, resistance has been reported in more than 500 insects, 50 of which transmit malaria parasites in humans [21, 25].

Insecticide resistance in malaria vector populations is widespread and covers all classes of insecticides recommended for public health use [26–30]. Pyrethroid resistance was first reported in Ivory Coast in 1993 [31]. Knockdown resistance (kdr) is currently the most common form of insecticide resistance. Outside Africa, kdr has been found in several malarial mosquito species, including *Anopheles stephensi* and *Anopheles culicifacies* [32, 33]. In Africa, kdr has been reported in a number of countries: in West Africa (Ivory Coast, Burkina Faso, Benin) [31, 34], Central Africa (Cameroon) [28], East Africa (Kenya) [35] and Southern Africa (South Africa and Zimbabwe) [36, 37].

Although insecticides have played a pivotal role in both agricultural and public health sectors, their widespread use has been linked to the development of insecticide resistance [38, 39]. The high frequency of kdr mutations in malaria vectors has been attributed to an extensive use of DDT to control agricultural pests in Africa [38] and Central America [40, 41]. Use of insecticides in IRS, and on bed nets and curtains has been associated with insecticide resistance in East Africa [35], Malaysia [42] and Sri Lanka [40].

Currently, there is insufficient information on the status of insecticide resistance in Zimbabwe. The available information may be underestimating the situation; the level of insecticide resistance may have in fact increased. Furthermore climate change, particularly the effects of temperature, may have influenced resistance [43, 44], but there is no concrete evidence of this as yet. We reviewed insecticide resistance data collected in the past 42 years in Zimbabwe and assessed the changes that have taken place. We then made recommendations for sustaining the progress made in malaria control through the use of insecticides.

## Review

### Materials and methods

We reviewed international peer-reviewed articles, published in English between 1972 and 2014, which assess the insecticide sensitivity status of mosquitoes in Zimbabwe. Selection for eligible studies was done through a literature search on MEDLINE® (PubMed), Google Scholar, Google and grey literature. The search terms were ‘insecticide resistance’, ‘sensitivity’, ‘temperature’, ‘vector mosquitoes’ and ‘Zimbabwe’. More literature was found using snowball sampling: that is identifying other papers listed from reference lists of initially identified articles. The abstracts of articles were read first, with the full articles read only if applicable. Papers were deemed eligible if they were about the insecticide sensitivity status of *An. gambiae s.l.* complex and *Anopheles funestus* mosquitoes in Zimbabwe.

## Results

### History of insecticide use in malaria control in Zimbabwe

Although DDT was the first insecticide to be used in IRS in Greece in 1947, by 1951 the insecticide had been effectively used in 22 countries [45]. In Zimbabwe, IRS began in 1949, using benzene hexachloride (BHC) [46]. However, some studies suggest that IRS was actually introduced in 1945, but launched in 1949 [47] (see Table 1). Published and grey sources state that between 1945 and 2003, BHC, DDT and pyrethroids were all used in IRS [48, 49; unpublished sources]. Following the discovery of BHC resistance in *An. arabiensis* mosquitoes in the Chiredzi district, BHC was replaced with DDT [48]. It should also be noted that although DDT was introduced in Zimbabwe in 1960, it was not extensively used until 1974, when resistance to BHC was reported [49]. Between 1976 and 1980, the control programme was disturbed by political unrest but soon after independence in 1980, IRS was resumed, using DDT.

**Table 1:** Summary of malaria control programmes using IRS in Zimbabwe between 1945 and 2004

Author	Year	Milestones in insecticide use in Zimbabwe
Mabaso et al., [47]	1945	IRS introduced
	1949	Programme launched
	1957–62	DDT and BHC used
	1972–73	BHC (equally effective as DDT but cheaper)
	1974–87	DDT (due to resistance to BHC)
	1988–2000	Deltamethrin and lambda-cyhalothrin (policy change)
UNEP, [50]	2004	DDT was reintroduced to complement pyrethroids
Unpublished sources/reports	1949–1960	BHC used for the countrywide malaria control programme, while dieldrin was used in sugar estates
	1960	DDT was used to complement BHC on a small scale
	1974–1976	DDT became a principal insecticide for malaria control
	1976–80	No insecticide used as spraying activities were disrupted by war of liberation
	1980–1987	Extensive use of DDT resumed
	1987–1991	DDT used, interchangeable with deltamethrin
	1991	DDT abandoned (decision to abandon was motivated by need to protect tobacco export)
	1991–2003	Only pyrethroids (deltamethrin, lambda-cyhalothrin and alpha-cypermethrin) were used
UNEP, [50]	2004	DDT was reintroduced to complement pyrethroids

From 1987 to 1991, DDT and deltamethrin were used alternately on malarial mosquitoes and tsetse flies. Other sources indicate that DDT was used between 1988 and 2000 [47]. However, DDT use was short-lived as environmentalists successfully lobbied for its ban in order to manage bed bug resistance. The insecticide’s lipophilic nature, which resulted in its accumulation in human adipose tissue, meat-eating birds and the environment at large, was another reason for its ban [51]. However, DDT was readopted in 2004 [50] to complement pyrethroids [37].

### Geographical distribution, causes and mechanisms of insecticide-resistant distribution over time

**Fig. 1:**
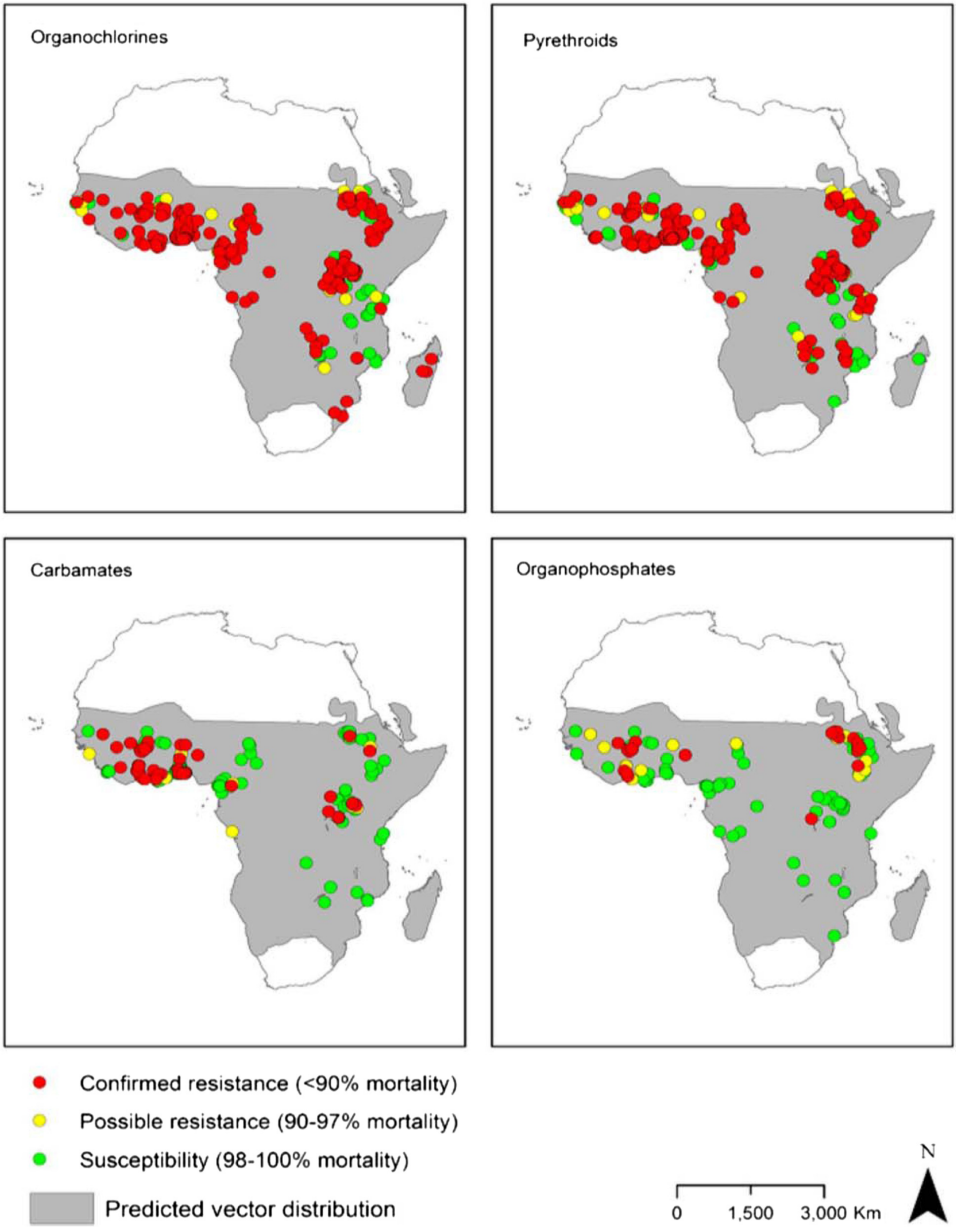
Distribution of insecticide resistance in *An. gambiae s.l.* mosquitoes between 2001 and 2012 [53]

An online literature search to review the resistance status of mosquitoes in the Afrotropical region, focusing on the period from 2001 to 2012, reaffirmed that malaria vectors were resistant to all insecticides recommended by the WHO for vector control in Africa. The same study reports resistance of *An. gambiae s.l* and *An. funestus* mosquitoes to organochlorines, carbamates and pyrethroids in Zimbabwe (see Fig. 1) [52].

### Case studies on insecticide resistance in Zimbabwe

In Zimbabwe, eight papers investigating insecticide resistance (1972–2014) have been published (see Table 2). These papers indicate that insecticide resistance levels have been changing over time. The first case of insecticide resistance (to BHC) was reported in Chiredzi in the early 1970s [48]. However, because Green’s original text (1972), which described this, could not be accessed, successive articles citing Green’s work, such as ones by Masendu et al. [54] and Munhenga et al. [37], were extrapolated on. In 1980, Crees reported on the susceptibility of mosquitoes in the areas of Chiredzi, Mtoko and Manjolo (unpublished data and not shown on Table 1). A study by Manokore et al. [49] documented that in the Gokwe region of Zimbabwe, there is an absence of insecticide resistance in mosquitoes to deltamethrin, alpha-cypermethrin, lambda-cyhalothrin and DDT. But after this study was conducted, insecticide resistance in *An. arabiensis* mosquitoes has been slowly spreading and increasing in intensity [54]. Munhenga et al. [37] further confirmed the presence of insecticide resistance to permethrin and DDT in *An. arabiensis* mosquitoes in Gokwe. Three papers reported insecticide resistance in *An. funestus* mosquitoes against organophosphates, pyrethroids and carbamates [5, 52, 55]. The two recent nationwide surveys contradict each other: The one conducted by the President’s Malaria Initiative (PMI) [5] reports insecticide resistance in *An. funestus* mosquitoes, while the other one by Lukwa et al. [56] disputes this as well as previous findings.

**Table 2:** Summary of studies that assessed insecticide resistance in malarial mosquitoes between 1972 and 2014 in Zimbabwe

Author [reference]	Objectives	Mosquito species studied	Study area	Method	Outcome
Knox et al. [52]	To introduce and demonstrate the usefulness of the online mapping tool IR Mapper	*An. gambiae*	African region (results presented are for Zimbabwe)	Systematic search of published peer-reviewed literature	*An. gambiae* and *An. funestus* were resistant to organophosphates and pyrethroids.
		*An. funestus*			
Lukwa et al. [56]	To conduct a nation-wide assessment of insecticide susceptibility in wild populations of *An. gambiae s.l.*	*An. gambiae* * s.l*	Thirteen (13) sentinel sites covering all malaria-endemic regions in Zimbabwe	All sites were sampled for resistance in malarial mosquitoes between 2011 and 2012.	No evidence of phenotypic resistance to any of the four insecticide classes in *An. gambiae s.l.* collected across different eco-epidemiology areas in Zimbabwe.
PMI Africa IRS. [5]	To determine insecticide susceptibility for malarial mosquito species from sentinel sites throughout Zimbabwe	*An. gambiae* * s.l*	Nine (9) sentinel sites in various provinces in Zimbabwe	WHO susceptibility tests were done using impregnated papers and test kits on wild caught *An. gambiae s.l.* and *An. funestus* mosquitoes.	*An. funestus* mosquitoes were resistant to pyrethroids (lambda-cyhalothrin and etofenprox).
		*An. funestus*			
Choi et al. [55]	To investigate the biological attributes of insecticide resistance and parasite infection rates that both impact on malaria vector control activities	*An. funestus*	Honde Valley	WHO susceptibility tests were done using impregnated papers and test kits on mosquitoes were collected between February and March 2014.	*An. funestus* populations were resistant to pyrethroids and carbamates.
Munhenga et al. [37]	To determine insecticide susceptibility of *An. arabiensis* using the WHO insecticide susceptibility method.	*An. arabiensis*	Gokwe	WHO susceptibility tests were done using impregnated papers and test kits on wild caught *An. arabiensis* and F1 progeny of the same mosquitoes.	Study confirmed the presence of permethrin and DDT resistance in *An. gambiae* mosquitoes in the Gwave area of Gokwe.
Masendu et al. [54]	To determine the distribution of malaria vectors in Zimbabwe together with the extent of insecticide resistance in different assemblages	*An. gambiae* Giles *s.s*, *An. arabiensis* Patton, *An. merus Dönitz* and *An. quadrinnulatus* Theobald (species A)*.*	Zimbabwe	National anopheline mosquito survey conducted between 1992 and 2002 at sites broadly categorised based on land use, patterns and location.	DDT resistance was detected in *An. arabiensis* collected from market gardens in Gokwe.
Manokore et al. [49]	To determine insecticide susceptibility of field caught *An. arabiensis* and F1 progeny reared from these field-caught females *An. arabiensis* Patton mosquitoes to WHO recommended insecticides	*An. arabiensis* Patton	Gokwe district in the Midlands province	Wild caught *An. arabiensis* mosquitoes were tested for insecticide sensitivity using the WHO susceptibility test method.	F1 progeny of field-caught females that were identified as *An. arabiensis* Patton were completely susceptible to deltamethrin, alpha-cypermethrin, lambda-cyhalothrin and DDT.
Green, [48]	Unknown	Unknown	Chiredzi district	Unknown mosquitoes were tested against BHC	Insecticide resistance to BHC reported in Chiredzi

The focus of research on insecticide sensitivity of mosquitoes has been Gokwe, where four studies have been conducted [37, 49, 54, 56]. This study site was chosen because of the presence of a National Institute of Health Research satellite station established to monitor malaria entomology in the area. Coetzee et al. [57] reiterated that early insecticide resistance distribution maps were limited, as they tended to reflect the distribution of entomologists rather than mosquito species. Perhaps this explains why this area has been so over researched.

**Fig. 2:**
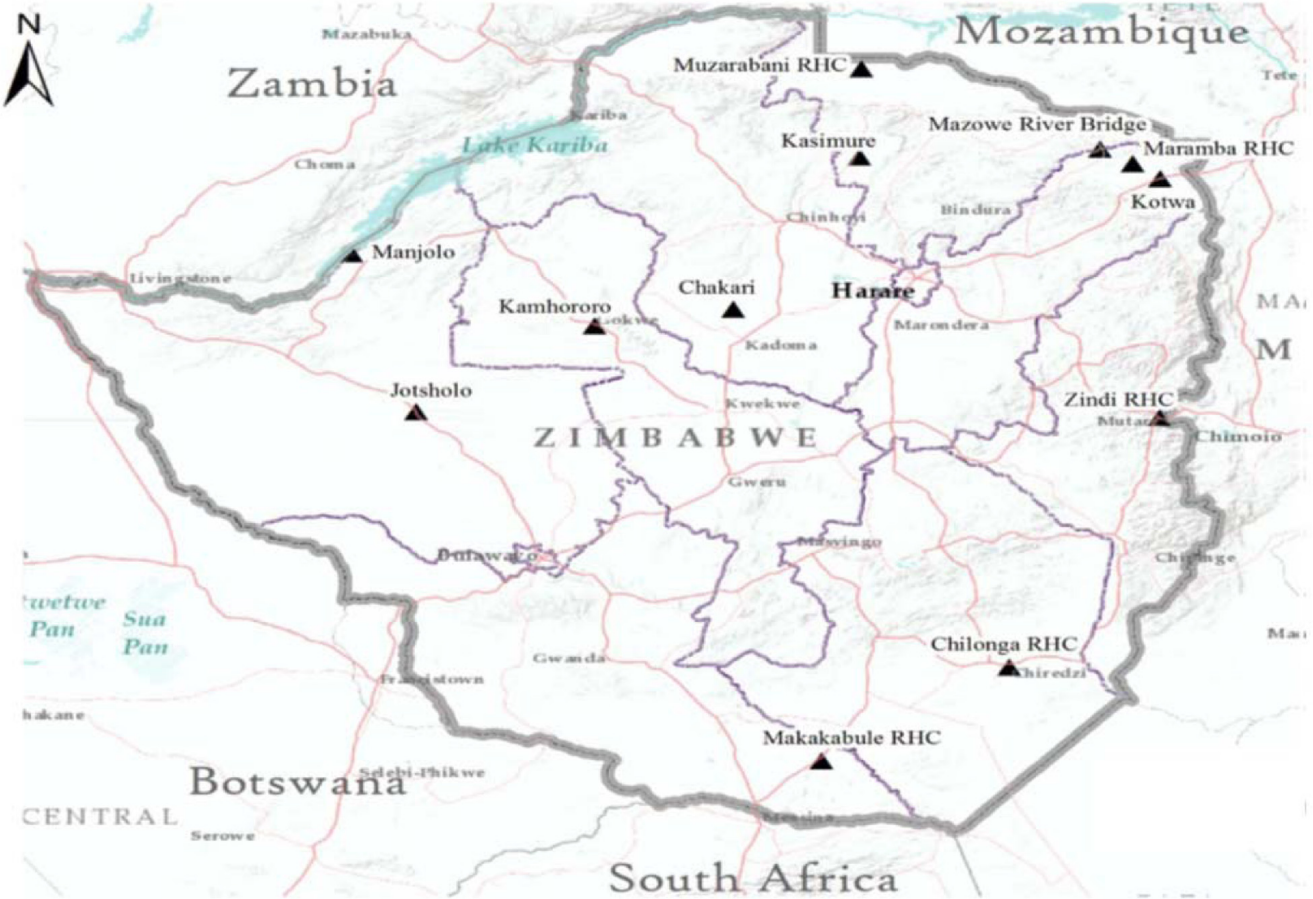
Map of Zimbabwe indicating the geographic location of the 12 insecticide-resistant monitoring sites (the *black triangles* represent villages where Lukwa et al. performed susceptibility tests) [56]

**Fig. 3:**
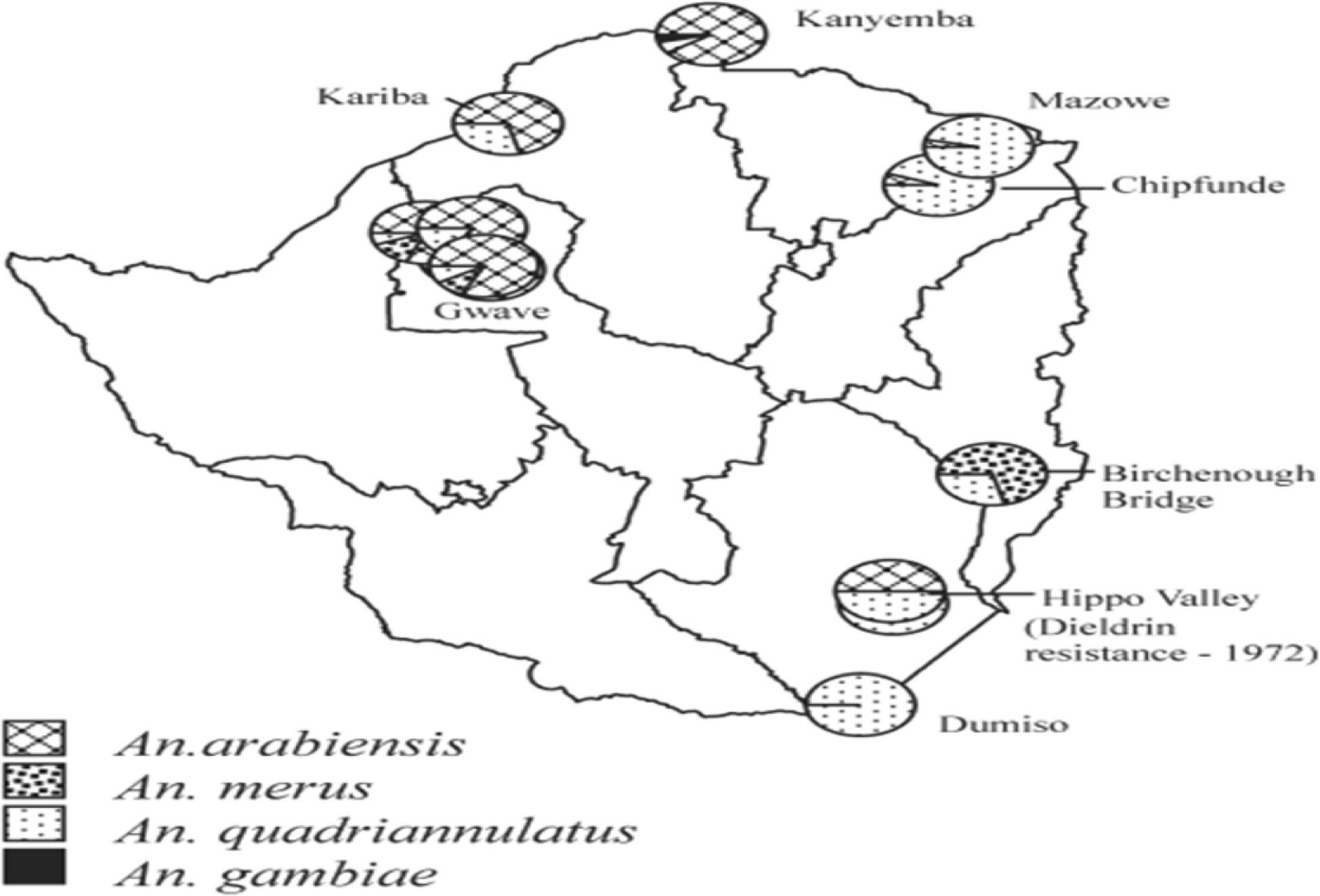
Sites where *Anopheles gambiae s.l.* mosquitoes were collected for susceptibility tests, showing Gwave (in Gokwe); sites where DDT resistance was detected in 2002; and Hippo Valley, where dieldrin resistance was detected in 1972 [54]

Some of the sites that have been sampled by Masendu et al. [54] and Lukwa et al. [56] are shown in Figs. 2 and 3. Contrary to the two recent studies [5, 56], previous studies observed insecticide resistance in mosquitoes in the Gwave area of Gokwe in 2008, and the first case of resistance in Hippo Valley in 1972 [37, 48, 54].

### Causes of insecticide resistance

#### Agricultural and public health practices

In Zimbabwe, agricultural practices have influenced the occurrence of resistance in malarial mosquitoes. Zimbabwe started using DDT as a pesticide in the agro-industry and for vector control programmes in 1969 [58]. The use of this pesticide resulted in a remarkable increase in agricultural production and improvements in human health as a result of controlling agricultural pests and arthropods that transmit animal and human diseases. However in 1972, DDT use was banned in the agro-industry because of public health concerns. Its use was eventually restricted to vector control programmes only [51]. A number of studies carried out after DDT was banned in Zimbabwe showed that the areas where DDT had been used (Esigodini, Nyanga, Kwekwe, Kadoma, Bulawayo, Harare and Kariba) were indeed polluted with organochlorine compounds [59, 60]. These insecticide pollutants might have exposed mosquitos to high or sublethal doses of the insecticides, which could have led to the development of insecticide resistance strains in malarial mosquitoes in some parts of the country.

### Climate change

As early as in the 1930s, Leeson [61] observed that mosquitoes were migrating from low to high altitude areas along river valleys in Zimbabwe. Ebi et al. [62] predicted that Zimbabwean highlands will be climatologically habitable to malarial mosquitoes by 2015 [13]. Similarly, Komen et al. [63] asserted that temperature was a critical factor in the transmission of malaria in the Limpopo province of South Africa [63], which shares a border with Zimbabwe. Mabaso et al. [64] also acknowledged that year-to-year variations in malaria incidences were mainly driven by climatic covariate, although this was not the only factor. In this context, it is imperative that we acquire more knowledge on mosquitoes’ responses and behaviours in the anticipated warmer climatic conditions.

### Main mechanism of resistance

Of the six studies that reported resistance [5, 37, 48, 52, 54, 55], only two determined the mechanism of resistance. Monooxygenase was responsible for resistance in *An. funestus* mosquitoes in Honde Valley [55]. Both East and West African kdr were identified as being responsible for resistance in *An. arabiensis* mosquitoes in Gokwe [37].

### Implications of malaria control

In Zimbabwe, the effect of resistance on vector control remains unknown. Although there is no scientific evidence to support the link, the Gokwe region, where resistance was reported on two occasions, has become one of the hubs of malaria transmission. There have also been sporadic outbreaks of malaria infection in the Honde Valley and Burma Valley. There have been no studies in Zimbabwe investigating the effect of resistance on malaria control, however, Corbel and N’Guessan [21] and Ranson et al. [19] have indicated that insecticide resistance is disruptive to malaria control programmes. The brief studies done in South Africa [36], Malawi [65], Burundi [66] and on the coast of Bioko Island, West Africa [67], all support the hypothesis that resistance is able to disrupt malaria control programmes. On the other hand, a study done in Zambia reports that insecticide resistance doesn’t interrupt malaria control [68].

## Discussion

This is the first paper to attempt to synthesise 42 years of data on insecticide resistance (from 1972 to 2014) in Zimbabwe. The increase in research work on the subject is evident by the higher number of papers being published on this topic; four papers in 2014 alone [5, 52, 55, 56]. A similar observation was made by Knox et al. [52], who noted an increase in the number of publications examining insecticide susceptibility and resistance in *Anopheles* mosquitoes in Africa. This could be due to researchers becoming increasingly concerned about the impact of insecticide resistance on malaria control programmes, in conjunction with their involvement in the NMCPs. Corbel and N’Guessan [21] and Ranson et al. [19] have indicated that insecticide resistance is disruptive to malaria control programmes in Africa.

The papers reviewed in this study describe fluctuations in the prevalence of resistance, with a non-uniform pattern, across Zimbabwe. In 2000, Manokore et al. [49] did not detect insecticide resistance in Gokwe. However, five and eight years later, two studies reported the presence of insecticides resistance among malarial mosquitoes in the same area [37, 54]. A nationwide survey conducted between 2011 and 2012 by Lukwa et al. [56] did not detect any insecticide resistance in malarial mosquitoes. However, Choi et al. [55] and PMI [5] reported resistance in samples collected between February and April 2014 in Honde Valley and Burma Valley, respectively, in *An. funestus* mosquitoes. Differences in the results of the three studies could be due to the differences in the sites sampled. Brogdon et al. [69] noted that the sites, which are only a few kilometres apart, were different not only because of the presence or absence of resistance, but also because of the varying levels of resistance and the dominant mechanisms responsible for resistance [70]. This indicates the importance of regularly sampling sentinel sites.

Despite strict rules governing insecticide use in the health and agro-industry sectors, the distribution of insecticide resistance in Zimbabwean mosquitoes seems to have been influenced by agricultural practices. The first case of insecticide resistance to BHC was reported in Chiredzi in 1972 [48]. In 2002, insecticide resistance to DDT in *An. gambiae sensu lato* mosquitoes was detected in Gokwe [54]. In 2008, resistance to pyrethroid (permethrin) and DDT was confirmed in Gwave, Gokwe [37]. Recently, resistance to carbamates and organochlorine was reported in *An. funestus* mosquitoes in Honde Valley [55] and to pyrethroids in Burma Valley [5]. Hippo Valley and the Triangle Estates are located in Chiredzi and are the sole sugar cane growers in the country. The estates have extensively used chemicals for pest control. The resistance of *An. gambiae* mosquitoes to DDT in Gokwe has also been attributed to the high usage of organochlorines by villagers, as well as a long history of DDT usage in this area for agricultural (especially cotton farming) and public health purposes, mainly tsetse and mosquito control [37, 54, 71]. Gipps [72] noted that Dicofol®, a chlorinated hydrocarbon which is used to control spider mite in cotton, cucurbits and tomatoes, contains 20 % DDT [72]. It is also believed that the water in Gokwe becomes contaminated when the pumps in the water pools are being cleaned [54]. Honde Valley and Burma Valley are also known for tea and banana farming by subsistence and commercial farmers [73].

Elsewhere in Africa, studies have attributed the high frequency of kdr mutations in malarial mosquitoes to extensive past use of DDT to control agricultural pests [38]. Persistent environmental contamination with organophosphate has also been a problem in Zimbabwe [58, 60, 74]. This stresses the importance of reviewing the regulations that govern the use of agricultural insecticides in Zimbabwe in order to curtail the spread of insecticide resistance.

The country’s malaria control programme needs to remain vigilant. A number of studies predicted that increased temperatures in conjunction with adequate rainfall would likely cause certain mosquito-borne infections to move to higher altitudes and latitudes [75], making some areas in Zimbabwe climatologically suitable for malaria transmission [62, 64]. None of the reviewed studies attempted to sample mosquitoes in middle veld areas. Temperature can influence the development of malaria parasites in the mosquito vectors, as well as in the development of the mosquito vectors themselves [76–78]. It can also influence the survival rate of the mosquito species; their survival rate at higher latitudes and altitudes; the alteration of their vectorial susceptibility to some pathogens; the rate of the vector population growth, host contact and feeding rate; as well as the seasonality of mosquito populations [79].

Moreover, temperature can alter the genetic structure, and enzyme and protein profiles of mosquitoes and other insects [80, 81]. For this reason, the insecticide sensitivity status of mosquitoes is inconclusive. Some studies suggest that high temperatures induce insecticide resistance in mosquitoes [43, 44]. In contrast, other studies note that high temperatures cause mosquitoes to become susceptible to insecticides [82]. In North-eastern United States, the *Wyeomyia smithii* mosquito species underwent genetic mutation in response to increased average land surface temperatures and spring coming earlier for two decades [83]. Although the *W. smithii* mosquito species is not a vector of human disease, it has similar physiological characteristics as the arbovirus species. This genetic alteration of *W. smithii* possibly points to similar changes occurring in malarial mosquitoes and hence underlines the need to investigate potential changes in malarial mosquitoes in Zimbabwe.

Ensuing field studies have indicated that insecticide resistance levels are dynamic and fluctuate throughout the malaria transmission season [84]. This observation may suggest that temperature might influence the development and levels of insecticide resistance, as each season has a unique average temperature.

The weakness of these studies is that they were based on different models, hypothesis and scenarios, and only a few mosquito species were investigated. Therefore, there is a need to conduct more studies in order to establish the effect of climate change, particularly temperature, on the development and distribution of insecticide resistance.

The current resistance situation in Zimbabwe is of public health concern as it confirms the notion that resistance in malarial mosquitoes now covers all classes of all chemicals approved for public health use [26–30]. Our review reports resistance to pyrethroids [5, 37, 55], organochlorines [54] and carbamates [55], but not organophosphates. Hence, periodic sampling of low velds and middle velds for malarial mosquitoes and testing for resistance may help in the early detection and monitoring of insecticide resistance.

Concerned with the current insecticide resistance situation, the Zimbabwe NMCP indicated, in the submission to The Global Fund’s new funding model for 2015–16, that organophosphates may be used in IRS. The NMCP’s plan for 2014 (October to December) was to conduct IRS using organophosphates in the areas with the highest resistance to pyrethroid. On the other hand, areas showing little or no pyrethroid resistance were to be sprayed using a mixture of pyrethroids and DDT [85]. Given that temephos (organophosphate) has already been used for larviciding in other parts of the country, it is possible that some mosquito species could now be resistant to organophosphates, the only class of insecticide in which resistance has not been reported in Zimbabwe.

The number of sites and frequency with which resistance monitoring should be conducted [86] has become a contentious issue. The number of sentinel sites that were sampled in the reviewed articles was relatively high in the 2005 survey [54] compared to the recent surveys [5, 56] in which there were lower, most probably due to a lack of resources. Hence, generalisation of these findings to the Zimbabwe situation needs to be done with care.

Furthermore, some studies did not sample key malaria areas. For instance, there were no sentinel sites sampled in the Kariba and Gwanda districts. In other instances, some provinces were under-represented, such as the Masvingo province in which only one rural health centre (Chilonga) was sampled, far too small to represent the entire province. The Chipinge district, which has been characterised by sporadic malaria outbreaks in previous years, was not represented in the survey [85]. This is not in line with the WHO guidelines, which state that insecticide resistance sentinel sites should be located in malaria-endemic areas with moderate to high malaria transmission rates. This means that study results not adhering to these guidelines need to be considered with caution.

It was also difficult to compare the recent countrywide survey results [5, 56] with the previous study done by Masendu et al. [54] due to variations in study areas (perhaps there were no sentinel sites back then). The minimum number of sampling sites should be determined considering the insecticide usage [19], location (rural and urban areas), and land use (where rice, cotton and vegetables are cultivated). It also needs to be informed by previous studies. In the recent two countrywide surveys, only one sentinel site in the urban area was sampled, yet Masendu et al. [54] observed the presence of *An. arabiensis* mosquitoes in the urban towns of Kwekwe, Chirundu, Kariba and Binga. Furthermore, the WHO criteria for the selection of insecticide resistance sentinel sites states that the sites should be established both in urban and rural settings [87].

Periodic seasonal sampling is recommended in order to detect seasonal resistance level variations; resistance is dynamic and wide fluctuations in resistance levels throughout the malaria transmission season have been reported [84]. This is important as resistance genes must not be allowed to build up because once they reach very high levels, strategies to restore susceptibility are unlikely to be effective [19]. Thus, regular seasonal monitoring of sentinel sites for resistance is vital in order to proactively prevent insecticides from affecting malaria control programmes.

Although the papers reviewed in this study had limitations, mainly due to a lack of resources, they do provide useful baseline information that can be used to conduct further studies on insecticide resistance, and how it might be influenced by climate change, in Zimbabwe.

## Conclusion

We do not conclusively know about the distribution of resistance in mosquitoes in Zimbabwe, and therefore more work needs to be done on this topic. Available information links insecticide resistance to agricultural activities, as insecticide resistance has been observed in areas where insecticides have been extensively deployed for agricultural and public health purposes. There are no reports of insecticide resistance in middle velds, as none of the studies have made attempts to sample these areas for insecticide resistance in mosquitoes.

The Zimbabwe NMCP needs to remain vigilant. It can do this by establishing sentinel sites in the middle veld, and by conducting periodic mosquito and resistance sampling in both low and middle velds. Areas where insecticide resistance has been detected need to be identified and the type of resistance needs to be elucidated. Sentinel sites need to be encompassing land used for different purposes, such as agriculture, and include rural and urban settlements.

Given the current insecticide resistance situation in the country, with resistance to three of the four classes of the WHO-recommended IRS insecticides (with the exception of organophosphates) being reported, rotation of insecticides to organophosphates remains the only option to restore insecticide susceptibility. Furthermore, usage of LLINs needs to be adapted to the resistance patterns and, most importantly, the country needs to develop a national resistance management plan.

## Abbreviations

BHC: Benzene hexachloride
DDT: Dichlorodiphenyltrichloroethane
GMEP: Global Malaria Eradication Programme
IRS: Insecticide residual spraying
Kdr: Knockdown resistance
LLIN: Long-lasting insecticide treated net
NMCP: National malaria control programme
PMI: President’s malaria initiative
WHO: World Health Organisation
